# Evaluating the Sensitivity of Wearable Devices in Posttranscatheter Aortic Valve Implantation Functional Assessment

**DOI:** 10.2196/65277

**Published:** 2024-11-08

**Authors:** Jinghui An, Fengwu Shi, Huajun Wang, Hang Zhang, Su Liu

**Affiliations:** 1 Department of Cardiac Surgery, the Second Hospital of Hebei Medical University, No 215, Heping West Road, Shijiazhuang, 050000, China, 86 0311-66002999

**Keywords:** aortic valve, implantation functional, wearable devices

I read with great interest the recent article by Eerdekens et al [1] titled “Cardiac Health Assessment Using a Wearable Device Before and After Transcatheter Aortic Valve Implantation: Prospective Study,” published in *JMIR mHealth and uHealth*. The study presented an innovative approach by using a wearable device to assess cardiac health outcomes before and after transcatheter aortic valve implantation (TAVI), addressing an important gap in the objective evaluation of functional improvement post TAVI.

While the findings of the study were significant, particularly the introduction of the Cardiac Energy Expenditure Slope (CEES) as a potential metric for assessing cardiovascular efficiency, there were some critical considerations that merited discussion.

First, the study’s conclusion that wearable device parameters, such as step count and total activity time, did not significantly change post TAVI, raised questions about the sensitivity of these devices in capturing subtle improvements in daily activity. It is well established that older populations, especially those undergoing TAVI, may not exhibit dramatic changes in physical activity due to a combination of frailty, preexisting comorbidities, and lifestyle factors [2-5]. However, the lack of significant change in these parameters might also reflect limitations in the wearable device’s ability to capture variations in physical activity that are clinically meaningful but subtle. For instance, improvements in quality of life and functional capacity may have manifested in ways that were not fully captured by metrics like step count alone.

Second, the study’s reliance on a 3-month follow-up period to assess post-TAVI outcomes could be seen as a limitation. While the authors argued that most patients reach full capacity by this time, it is possible that some patients might show delayed improvements in physical activity and cardiovascular efficiency. Extending the follow-up period to 6 months or even a year could have provided a more comprehensive view of the long-term impact of TAVI on patient activity levels and cardiac health.

Additionally, the introduction of CEES as a novel metric was intriguing, yet its application and utility needed further validation in larger, diverse cohorts. The metric’s correlation with traditional measures of functional improvement, such as the 6-minute walking test, and its predictive value in long-term outcomes post TAVI, should be explored in future studies. This would help establish CEES as a reliable tool in both clinical practice and research settings.

In conclusion, while the study by Eerdekens et al [1] contributed valuable insights into the use of wearable devices for cardiac health assessment, it also highlighted the need for further research to optimize these tools for older, comorbid populations. Continued exploration into novel metrics like CEES and longer follow-up periods could enhance our understanding of post-TAVI recovery and guide personalized treatment strategies.

## Abbreviations

CEES: Cardiac Energy Expenditure Slope
TAVI: transcatheter aortic valve implantation
